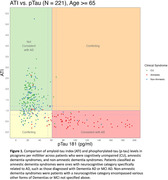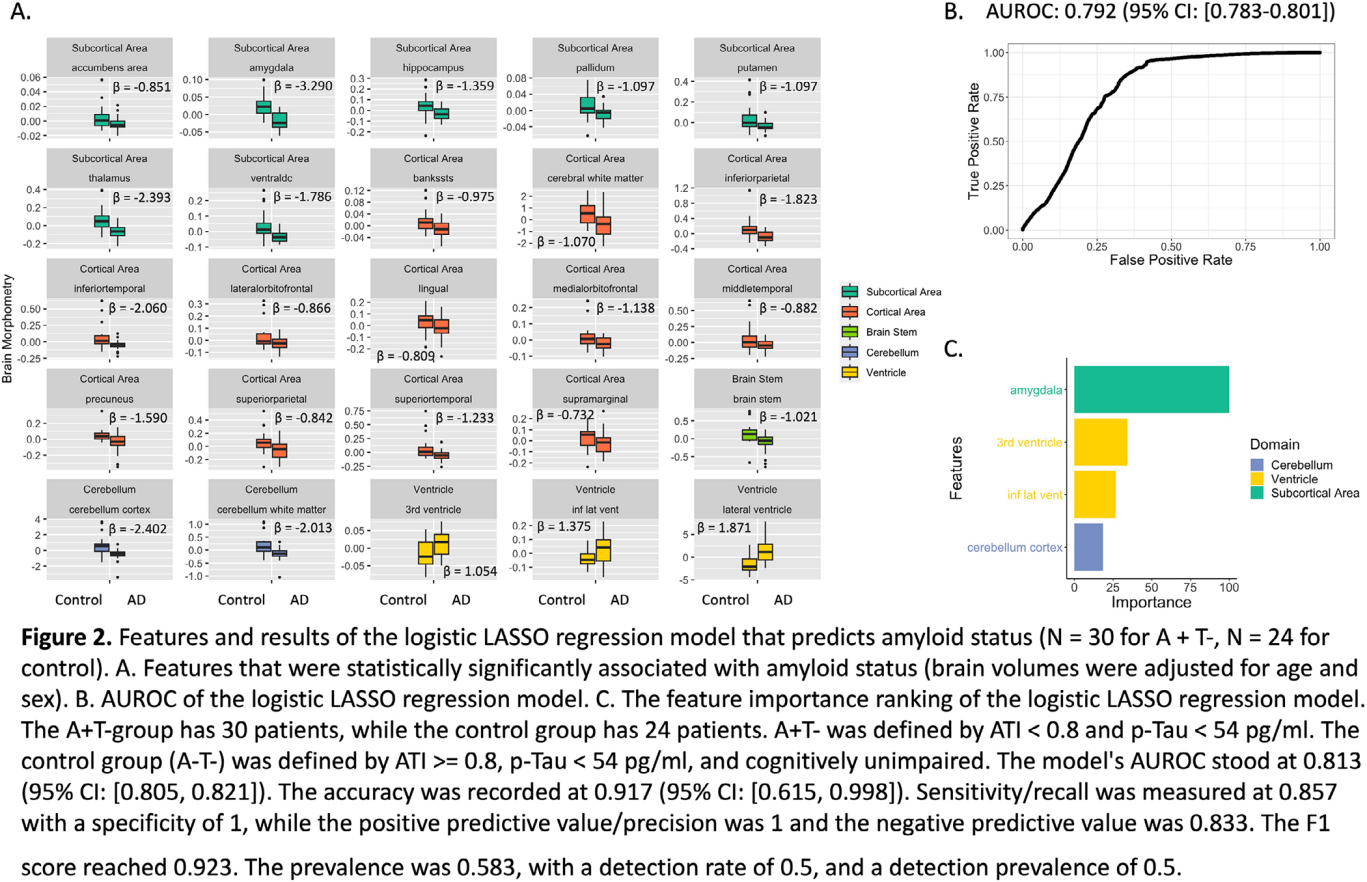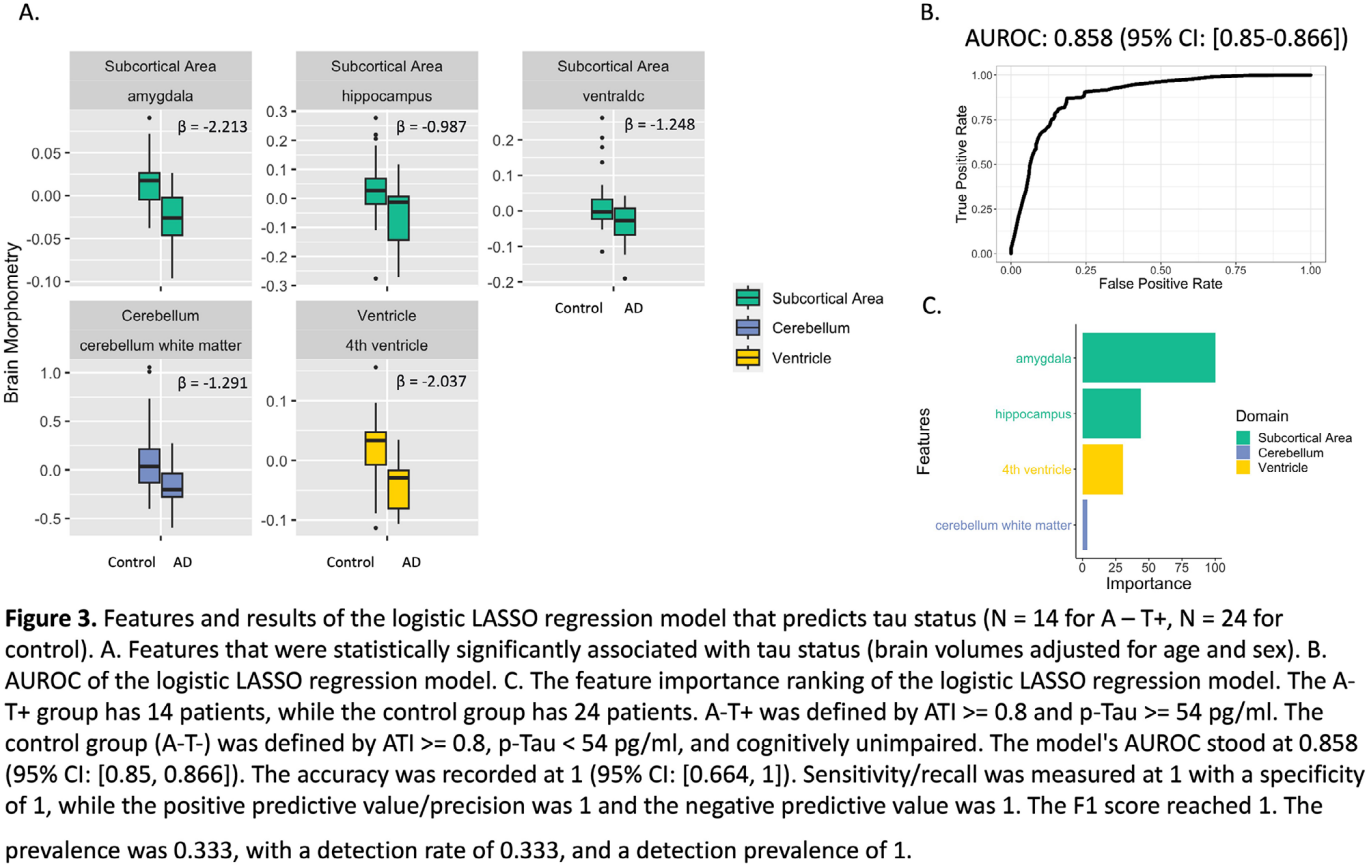# Association of brain MRI signatures with Alzheimer Disease CSF biomarkers

**DOI:** 10.1002/alz.092833

**Published:** 2025-01-09

**Authors:** You Cheng, Karthik Gopinath, Benjamin Billot, Juan Eugenio Iglesias, Alexandra Touroutoglou, Pia Kivisäkk, Chao‐Yi Wu, Hiroko H Dodge, Bradley T. T Hyman, Steven E Arnold, Sudeshna Das

**Affiliations:** ^1^ Massachusetts General Hospital, Harvard Medical School, Boston, MA USA; ^2^ Massachusetts General Hospital, Charlestown, MA USA; ^3^ Massachusetts Institute of Technology, Cambridge, MA USA; ^4^ Martinos Center for Biomedical Imaging, Massachusetts General Hospital and Harvard Medical School, Boston, MA USA; ^5^ Centre for Medical Image Computing, University College London, London UK; ^6^ Computer Science and Artificial Intelligence Laboratory, Massachusetts Institute of Technology, Cambridge, MA USA; ^7^ Department of Neurology, Harvard Medical School, Boston, MA USA; ^8^ Massachusetts General Hospital, Boston, MA USA

## Abstract

**Background:**

The relationship between CSF measures of Alzheimer disease (AD) pathologies and their neurodegenerative signatures is not fully understood. This study seeks to employ machine learning approaches on clinical MRI data to identify patterns associated with amyloid and tau, aiming to guide diagnosis and therapeutic interventions.

**Method:**

We selected brain volumes that differed significantly between AD pathology and control groups. Then we utilized logistic LASSO regression to identify and compare neurodegenerative signatures associated with amyloid and tau burdens using selected brain volumes. Our methods were applied to a dataset that included clinical MRI scans from patients who also had cerebrospinal fluid (CSF) biomarkers assessed within a year of the MRI scan (Figure 1). Model performance was measured using the cross‐validation area under the receiver operating characteristic curve (AUROC), as well as the accuracy, sensitivity, and specificity in a 25% held‐out test set.

**Result:**

For amyloid status, among 30 amyloid‐positive, tau‐negative (A+T‐) patients and 24 cognitively unimpaired A‐T‐ controls, we identified 25 significant features associated with amyloid status, including subcortical areas (e.g., amygdala, thalamus, hippocampus), cortical areas (e.g., inferior temporal cortex, inferior parietal cortex, precuneus), ventricles (lateral ventricle, inferior lateral ventricle, and the 3^rd^ ventricle), cerebellum (cerebellum white matter and cerebellum cortex), and brain stem (Figure 2A). Our model with these features yielded an AUROC of 0.792 (95% CI: [0.783, 0.801]), and a sensitivity of 0.857 at specificity of 1 (Figure 2B). In the assessment of tau status, where the amyloid‐negative, tau‐positive group (A‐T+) included 14 patients and the control group comprised 24. The volumes showed significant difference associated with tau status, including subcortical areas (amygdala, ventral diencephalon, hippocampus), the 4^th^ ventricle, and cerebellum white matter (Figure 3A). The model achieved an AUROC of 0.858 (95% C.I. [0.85, 0.866]), with a sensitivity of 1 at specificity of 1 (Figure 3B and C).

**Conclusion:**

The study underscores the potential of using widely available clinical MRI images to measure AD and associated pathologies in the brain, which hold significant promise for guiding treatment interventions.